# Noble Metal-Free Light-Driven Hydrogen Evolution Catalysis in Polyampholytic Hydrogel Networks

**DOI:** 10.1021/acsami.4c04045

**Published:** 2024-05-03

**Authors:** Tolga Ceper, Daniel Costabel, Daniel Kowalczyk, Kalina Peneva, Felix H. Schacher

**Affiliations:** † Institute of Organic Chemistry and Macromolecular Chemistry, Friedrich Schiller University Jena, Humboldtstraße 10, D-07743 Jena, Germany; ‡ Jena Center for Soft Matter (JCSM), Friedrich Schiller University Jena, Philosophenweg 7, D-07743 Jena, Germany; § Center for Energy and Environmental Chemistry Jena (CEEC), Friedrich Schiller University Jena, Philosophenweg 7a, 07743 Jena, Germany; ∥ Institute of Chemical Engineering, Ulm University, Albert-Einstein-Allee 11, 89081 Ulm, Germany

**Keywords:** hydrogen evolution catalysis, noble metal-free, polyampholyte, hydrogel, scaffold, immobilization

## Abstract

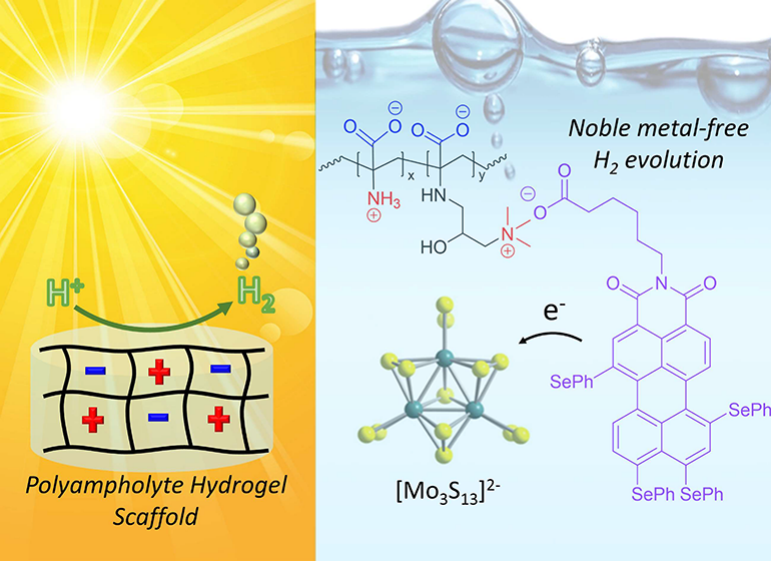

Future technologies to harness solar energy and to convert this into chemical energy strongly rely on straightforward approaches to prepare versatile soft matter scaffolds for the immobilization of catalysts and sensitizers in a defined environment. In addition, particularly for light-driven hydrogen evolution, a transition to noble metal-free photosensitizers and catalysts is urgently required. Herein, we report a fully organic light-harvesting soft matter network based on a polyampholyte hydrogel where both photosensitizer (a perylene monoimide derivative) and a H_2_ evolution catalyst ([Mo_3_S_13_]^2–^) are electrostatically incorporated. The resulting material exhibits sustained visible-light-driven H_2_ evolution in aqueous ascorbic acid solution, even at rather low loadings of photosensitizer (0.4%) and catalyst (120 ppm). In addition, we provide initial insights into the long-term stability of the hybrid hydrogel. We believe that these results pave the way for a generalized route toward the incorporation of noble metal-free light-driven catalysis in soft matter networks.

## Introduction

The efficient and sustainable production of fuels and chemicals by utilizing abundant and renewable sources is inevitable due to global warming.^1^ Addressing the growing demand for sustainable energy solutions, artificial photosynthesis emerges as a promising concept.^2^ Inspired by the natural process in plants, it harnesses solar power to convert abundant resources such as water (H_2_O) and carbon dioxide (CO_2_) into valuable chemicals such as hydrogen (H_2_), carbon monoxide (CO), and hydrocarbons.^3^ This innovative approach integrates two key components: a light-harvesting unit and a catalytic site. Together, these elements mimic the intricate mechanisms of photosynthesis, offering a sustainable pathway toward producing renewable fuels and chemicals.^4^ Among them, hydrogen serves as an appealing green energy carrier, finding application as a direct fuel in hydrogen fuel cells and as a feedstock in various chemical processes.^5,6^ This concept has triggered extensive research in photocatalytic hydrogen evolution reaction (HER), resulting in the discovery of numerous catalysts such as noble metals,^7^ metal oxides,^8^ metal–organic frameworks,^9^ and recently thiomolybdates.^10^ Specifically, the model compound [Mo_3_S_13_]^2–^ represents an earth-abundant alternative to conventional catalysts (CAT), exhibiting impressive turnover numbers (>40,000) and high turnover frequencies (>150 min^–1^) in light-driven HER.^11,12^ The catalytic activity of [Mo_3_S_13_]^2–^ under visible light relies on the presence of molecular photosensitizers (PSs) that capture incoming light energy and transfer electrons to the catalytic center.^12^

Efficient light absorption and appropriate energy level alignment between PS and CAT are crucial for maximizing the conversion of solar energy into chemical bonds, thus enhancing the overall efficiency of hydrogen evolution catalysis.^13^ Various compounds have been investigated for their potential as PSs in visible light-driven HER. These include metal complexes,^14^ conjugated polymers,^15^ quantum dots,^16^ and organic dyes.^17,18^ Rylene-based dyes, such as perylene diimide (PDI), perylene monoimide (PMI), and their derivatives, have emerged as a promising class of PSs due to their noble metal-free nature, excellent visible light absorption, and high photostability.^19,20^ They can form light-absorbing supramolecular materials (e.g., nanorods) via self-assembly, driven mainly by pi–pi electronic interactions, enhancing the visible light absorption range of HER catalysts such as Pt/g-C_3_N_4_ and Pt/TiO_2_.^21,22^ Alternatively, PMI can function as a molecular PS when modified at the “bay” region, altering the pi-system to prevent aggregation.^23^ Thio- and selenophenoxy substituents at this region allow tuning of photophysical properties, including bathochromic shift and increased electron density, and the generation of long-lived triplet states. While solubility in organic solvents is enhanced, using them as a PS in water-based systems is challenging. Recent studies demonstrate that PMI derivatives with these modifications can sensitize [Mo_3_S_13_]^2–^ clusters for stable HER under visible light, facilitated by poly(dehydroalanine)-*graft*-poly(ethylene glycol) (PDha-*g*-PEG) graft copolymers as solubilizing templates.^24^

Using soft matter matrices like polymers,^25,26^ membranes,^27^ and micelles^28^ for light-driven catalysis is appealing. These matrices not only address the limited solubility of many PSs in aqueous environments but also allow for a defined spatial arrangement of the PS and catalyst, often boosting catalytic activity.^29^ In recent years, researchers working in the field of photocatalytic H_2_ evolution have also become increasingly interested in hydrogels due to their appealing features including the 3D accessibility of embedded molecules and excellent water uptake.^30,31^ Furthermore, they offer attractive solutions for subsequent postprocessing tasks such as handling, transportation, and washing if necessary, as well as recyclability.^32^ Notably, these macromolecular networks are typically formed by the self-assembly of either the PS or the catalyst.^30,33−35^ In contrast, only a few examples have been reported that utilize a preformed hydrogel as scaffold. For instance, PMI ribbons are entrapped inside polyelectrolyte hydrogels formed by free radical polymerization of a cross-linker, acrylamide, and charged comonomers such as 3-acrylamidopropyl-trimethylammonium chloride (APTAC) or 2-acrylamido-2-methyl-1-propanesulfonic acid sodium salt (AMPS).^36^ However, the performance of these systems often relies on the self-assembly process of PMI molecules within the hydrogel network. Another prime example described by Okeyoshi et al. involves a H_2_-evolving gel obtained by incorporating a HER catalyst into a cross-linked network comprising comonomers derived from [Ru(bpy)_3_]^2+^ (bpy = 2,2′-bipyridine) and viologen.^37,38^ While this gel system offers flexibility in usage and potential adaptation to catalytic systems for O_2_ production,^39^ the demanding preparation of monomers involved in the catalytic process (e.g., light-harvesting, electron relay) poses a significant challenge. Inspired by this, we have reported a polyampholyte hydrogel designed for immobilizing a PS and CAT only via electrostatic attachment, demonstrating the capability for both visible light-driven HER and water oxidation.^40,41^ These networks, based on polydehydroalanine (PDha), form hydrogels with dynamic charges arising from ionizable groups (−NH_2_ and −COOH). Therefore, they can accommodate both positively and negatively charged guest molecules, making them suitable matrices for various PS/catalyst combinations through attractive electrostatic interactions. Moreover, these networks can be easily and further functionalized.^42,43^

We herein aim for a heterogeneous system capable of noble metal-free visible light-driven HER catalysis using polyampholyte hydrogels based on PDha. We immobilized two molecular building blocks, an organic PS (derived from PMI) and a molybdenum CAT ([Mo_3_S_13_]^2–^), via attractive electrostatic interactions. Both molecular building blocks feature negative charge and are bound to cationic sites within the hydrogel which were introduced to PDha before cross-linking. By electrostatic attachment, we aim for precise control over the arrangement and distribution of the photosensitizer molecules within the hydrogel matrix, optimizing their exposure to incident light, while enhancing the stability of the composite through electrostatic interactions between the PS and the hydrogel. This approach may offer advantages in terms of long-term performance and sustained hydrogen evolution activity over extended periods, particularly when compared to alternative methods. As the PDha hydrogels can also be swollen in organic solvents, the attachment of the PS was realized in DMF, followed by the stepwise addition of [Mo_3_S_13_]^2–^ to an aqueous ascorbic acid solution. The resulting hybrid hydrogels featured prolonged H_2_ evolution under visible light irradiation. We describe initial findings regarding the stability and reactivity of the hydrogel scaffold, together with a discussion of present restrictions and prospective paths for advancement toward more stable and technologically relevant advanced systems.

## Materials and Methods

### Materials

All chemicals were purchased from Sigma-Aldrich Chemie GmbH (Münich, Germany) and used as received. Analytical grade solvents were purchased from Sigma-Aldrich Chemie GmbH (Münich, Germany) or VWR International GmbH (Darmstadt, Germany). *tert*-Butoxycarbonylaminomethyl acrylate (*t*BAMA) was synthesized according to the literature.^44^ BlocBuilder and SG-1 were synthesized following a reported procedure.^45^ 1,7,9,10-Tetraselenophenoxy perylene monoimide (PMI) was synthesized according to a reported protocol.^24^ (NH_4_)_2_[Mo_3_S_13_]·2H_2_O was prepared as reported earlier.^40^

### Synthesis of PDha-*g*-GTMAC Hydrogels

P(Dha-*co*-AMA) was synthesized by cleavage of Boc and methyl ester groups on P*t*BAMA (*M*_n_ of 18.000 g/mol) synthesized by nitroxide-mediated polymerization of *t*BAMA as described in an earlier report.^40^

#### Synthesis of PDha-*g*-GTMAC

P(Dha_0.9_-*co*-AMA_0.1_) (200 mg) and glycidyltrimethylammonium chloride (GTMAC) (1 equiv per monomer unit) were dissolved in 0.1 M KOH solution (20 mL each, pH 13). The resulting solution was held in an oil bath at 60 °C under constant stirring for 96 days. Afterward the reaction mixture was neutralized by adding aqueous HCl (0.5 M) until pH 7. The crude product was dialyzed against DI water for 48 h with at least four water changes and finally freeze-dried to get a white polymer powder.

#### Chemical Cross-Linking of PDha-*g*-GTMAC

PDha-*g*-GTMAC (30 mg) was dissolved in 0.9 mL of 0.1 M KOH overnight at 60 °C under stirring in a 5 mL glass vial. Poly(ethylene glycol) glycidyl ether with a *M*_n_ of 2000 g·mol^–1^ (175 mg) as cross-linker was then added to the solution, purged by argon, and finally held in an oil bath at 60 °C for 16 h. The resulting hydrogel was soaked in 20 mL of DI water for 48 h with at least four water changes to remove the unreacted polymers and cross-linkers. Hydrogels in water at pH 7 were freeze-dried to get a colorless solid before the characterization.

### Gel Content and Composition Analysis

Gel content of the hydrogel was determined by weight loss of the hydrogel during the dialysis. Accordingly, hydrogel composition was first examined by analyzing the chemical composition of the dialysate using quantitative ^1^H NMR spectroscopy. To this end, the mixture of precursor polymer and cross-linker in DI water at various ratios was prepared. Then the resulting solution was freeze-dried, the obtained powder was dissolved in D_2_O, and ultimately a calibration curve was drawn according to the ratio of ^1^H NMR signals. Second, the elemental contents at different PDha-*g*-GTMAC-to-cross-linker ratios were calculated by theoretical chemical analysis. Experimentally obtained [C]/[N] ratios were compared with theoretical values to estimate the PDha-*g*-GTMAC fractions in the hydrogels.

### Degree of Swelling

Degree of swelling (DS) was calculated by measuring the weights of swollen hydrogels equilibrated in deionized (DI) water and/or NaCl solution and the corresponding dried gel on a preweighed clock glass. Gels were dried overnight on a hot Teflon plate at 90 °C in air. Surface water or solution on a fully swollen gel was excluded by using a soft tissue before weighing. Triplicate measurements were performed to take into account the errors in the measurements. The swelling degree of hydrogels was calculated according to eq 1.
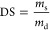
1

### Preparation of Organic Light Harvesting Soft Matter Networks

PMI-Se-COOH (10.5 mg) was initially dissolved in 10 mL of alkaline water with NaOH (1 equiv per −COOH unit) and then freeze-dried in small portions according to the target concentration. The resulting purple-blue solid was dissolved again in 2 mL of DMF. Freeze-dried hydrogels with ∼30% PDha-*g*-GTMAC were placed in the prepared PMI-Se-COOH solution at room temperature for 16 h. The supernatant was colorless and analyzed by UV–vis spectroscopy to determine the adsorbed dye amount. The soft network was obtained as a purple-blue solid.

### Visible Light-Driven Hydrogen Production

Hydrogen production was performed using a prepared soft network combined with a HER catalyst under visible light. In a typical experiment, the organic light-harvesting soft network (PDha-*g*-GTMAC content is 30%, PMI-Se-COOH content is 0.4%) was placed in 2 mL of ascorbic acid solution (1 M) at pH ≈ 5.5 adjusted by NH_4_OH solution in a 5 mL glass GC vial. Na_2_[Mo_3_S_13_]·5H_2_O dissolved in DMF (0.1 mg/mL) was then added to the reaction vial, followed by heating at 50 °C for 15 min for a better catalyst diffusion. The resulting system was purged with argon, and the vial was sealed with a septum cap and eventually irradiated from the bottom with a blue LED light (λ_max_ = 453 nm, incident photon flux = 0.751 μmol·s^–1^, operating current = 960 mA) in a custom-built, air-cooled photoreactor^46^ (utilizing a sample holder for 6–8 vials placed at 5 cm distance from the bottom) on a platform shaker. Hydrogen production (H_2_ accumulated in the headspace) was quantified by an Agilent A7820A gas chromatograph (GC) with a molecular sieve A5 column. Detection is afforded by a thermal conductivity detector with nitrogen carrier gas. The catalyst stock solution was freshly prepared each day of analysis.

For PS leaching, PS@PDha-*g*-GTMAC was held in a 1 M ascorbic acid (AA) solution at pH ≈ 5.5 at room temperature under stirring for 24 h and then removed, the catalyst was added into the remaining solution, and the solution was irradiated for 24 h after purging with argon. Hydrogen evolution was quantified by GC, and the remaining solution was analyzed by UV–vis spectroscopy.

The photonic efficiency (PE) was calculated based on eq 2.^47^ The performed PE calculations are based on the photon flux incident to the reaction solution, derived from the photonic characterization data of the modular photoreactor.^46^
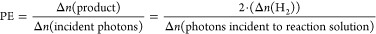
2

### Nuclear Magnetic Resonance (NMR) Spectroscopy

^1^H and ^13^C NMR spectra were performed on a Bruker AC 300 MHz using CDCl_3_ and D_2_O/NaOD as solvents at a temperature of 298 K. The spectra were referenced by using the residual signal of the deuterated solvent. ^1^H NMR spectra were recorded with a 4 mm high-resolution magic angle spinning (HRMAS) probe (PH HR MAS 500 S1 CHD 4 G) with a freeze-dried gel sample swollen in CDCl_3_ (semisolid state) and a MAS frequency of 4 kHz at 297 K.

### Size Exclusion Chromatography (SEC)

SEC traces in THF were measured using an Agilent 1260 Infinity system equipped with a 1260 IsoPump (G1310B), a 1260 ALS (G1310B) autosampler, and three consecutive PSS SDV, 5 μm, 8 × 300 mm, columns. The eluent flow rate was set to 1 mL·min^–1^, and the column oven was set to 40 °C. Signals were detected using a 1260 DAD VL (G1329B) and a 1260 RID (G1315D) detector. The system was calibrated using PMMA (Mp: 2.2M-800 Da) standards.

### FTIR Spectroscopy

Infrared spectra were measured on a PerkinElmer Frontier FT-IR/NIR spectrometer equipped with a Golden Gate ATR unit from Sepcac. The spectra were recorded using 40 scans at a resolution of four wavenumbers between 4000 and 400 cm^–1^.

### Elemental Analysis

Elemental analysis was performed on a Vario EI III elemental analyzer.

### UV–Vis Spectroscopy

Absorbance spectra were recorded on an Agilent Cary 60 instrument in a plastic cuvette with a path length of 10 mm at room temperature. The absorbance was measured in a range.

### Scanning Electron Microscopy (SEM)

SEM micrographs were acquired on a Zeiss Sigma VP instrument at an acceleration voltage of 14 kV with an SE2 detector.

## Results and Discussion

**Scheme 1:**
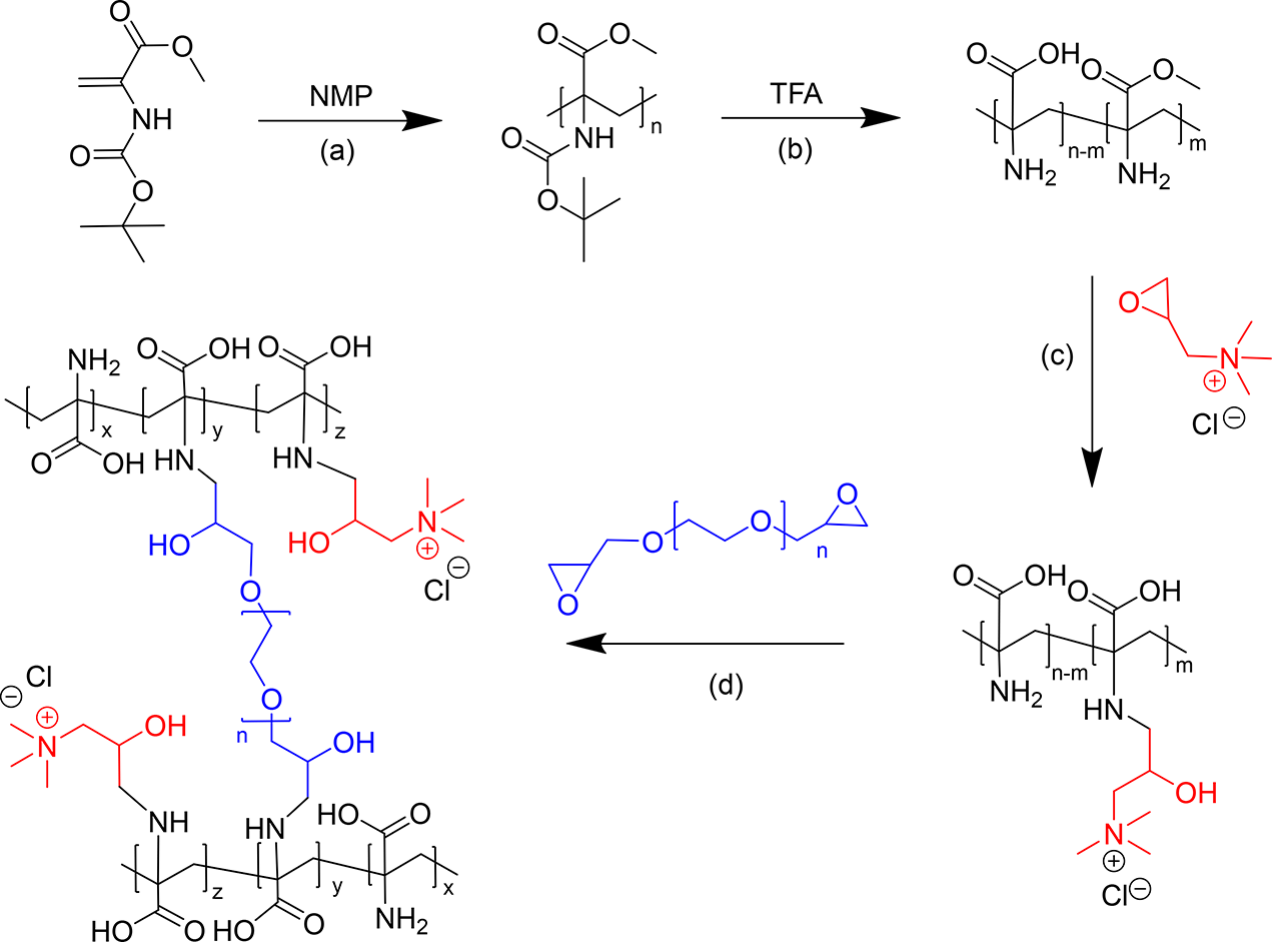
Synthetic Pathway of PDha-*g*-GTMAC-Based Hydrogel Formation Including (a) Polymerization of *t*BAMA, (b) Deprotection of P*t*BAMA, (c) Post-polymerization Modification of P(Dha-*co*-AMA), and (d) Chemical Cross-Linking of PDha-*g*-GTMAC

The chemical cross-linking of poly(dehydroalanine) allows us to obtain pH-responsive, transparent, and self-supporting hydrogels featuring a high density of pH-dependent charges, and these hydrogels were already successfully utilized as scaffold matrix for immobilizing [Ru(bpy)_3_]^2+^ photosensitizers and H_2_ or O_2_ evolution catalysts.^40,41^ In this work, we integrate an organic photosensitizer derived from PMI into PDha-based hydrogels bearing quaternary ammonium side chains to obtain a fully organic light-harvesting hybrid hydrogel. To this end, we used a PMI derivative with a negatively charged anchoring group for attachment and modified PDha before cross-linking to increase the adsorption capability. The hydrogel synthesis was done in three consecutive steps involving polymer synthesis, postpolymerization modification, and chemical cross-linking (Scheme 1). P(Dha-*co*-AMA) was obtained by deprotection of P*t*BAMA with a molecular weight (*M*_n_) of 18.000 g·mol^–1^ (*Đ* = 1.87) synthesized by nitroxide-mediated polymerization (NMP) of *t*BAMA as described in our earlier report (Figure S1, see ^1^H NMR spectra).^44^ P(Dha-*co*-AMA) contained around 10% of methyl ester protecting groups, which can be hydrolyzed during the postpolymerization modification, yielding PDha.^48^

PDha bears reactive amines, which allow the introduction of a variety of functional groups,^48^ and here these groups were modified with GTMAC by nucleophilic ring-opening of the epoxide, where the reaction protocol was adopted from our earlier reports.^48−50^ The successful modification and the presence of GTMAC were confirmed via ^1^H and ^13^C NMR and FT-IR spectroscopy (Figures S2–S4, see SI for detailed characterization). The degree of functionalization (DoF) of PDha-*g*-GTMAC was determined from ^1^H NMR spectra by peak areas of the backbone and the new side-chain protons (Figure S2). Grafting was carried out at varying P(Dha-*co*-AMA)-to-GTMAC ratios and reaction times, resulting in PDha-*g*-GTMAC with a DoF ranging from 23% to 99% (Table S1). These graft copolymers were subsequently used in the synthesis of polyampholytic hydrogels with an optimum ratio of cationic anchoring sites to additional cross-links.

**Figure 1:**
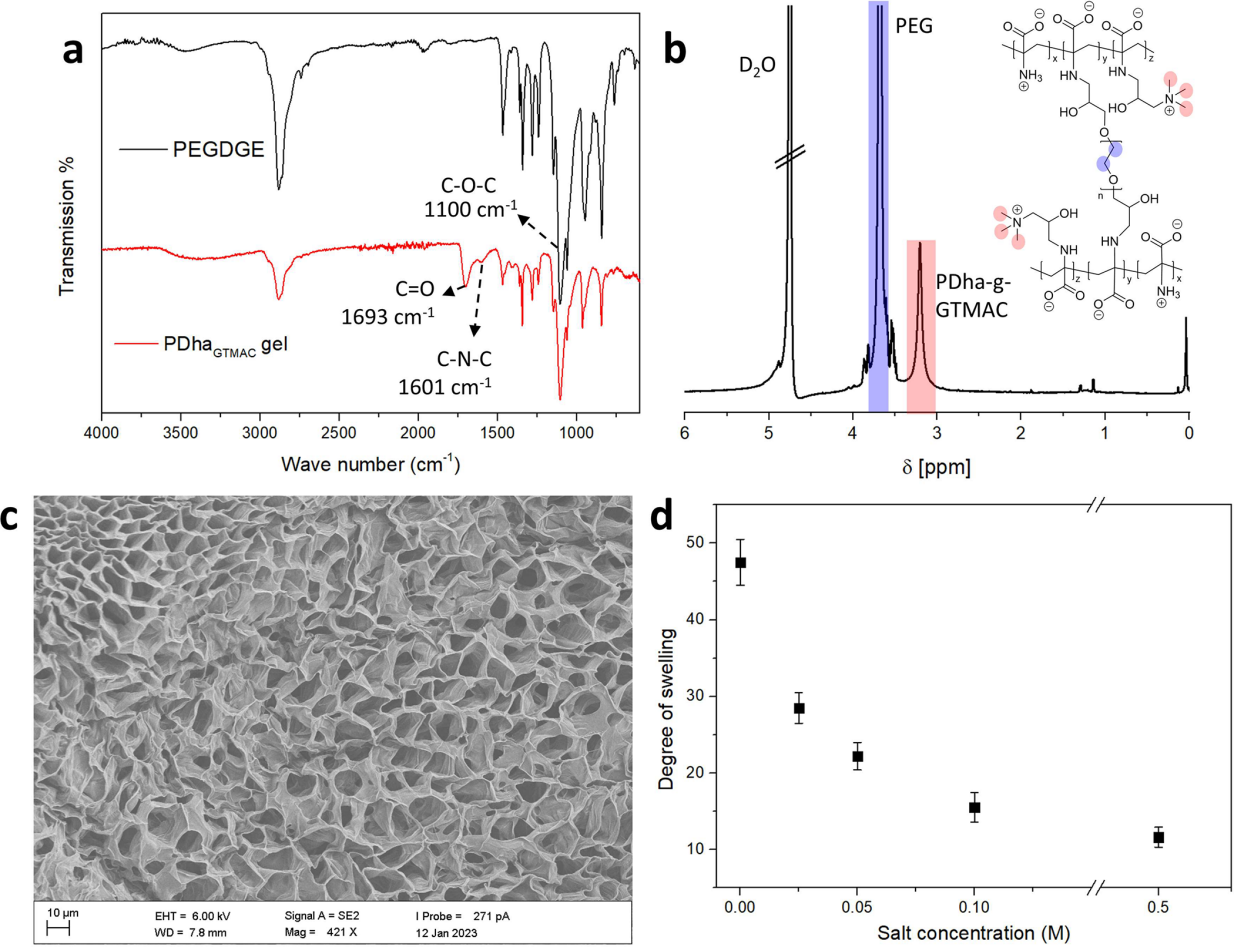
Comparison of FT-IR spectra of the gel and the cross-linker PEGDGE (A), ^1^H HR-MAS NMR spectrum of a PDha-*g*-GTMAC hydrogel (B), SEM micrograph of the freeze-dried gel (C), and the degree of swelling of a PDha-*g*-GTMAC hydrogel at varying salt concentrations (D).

The chemical cross-linking of PDha-*g*-GTMAC was realized using poly(ethylene glycol) diglycidyl ether (PEGDGE) with *M*_n_ ranging from 500 to 2000. Gelation was achieved after 18 h at 70 °C using PEGDGE (*M*_n_ = 2000) at a 0.69 amine-to-epoxide ratio and a 208.5 mg·mL^–1^ prepolymer mixture concentration in 0.1 M KOH solution, resulting in a transparent and self-supporting hydrogel. It should be noted that this approach for gelation was successful for a DoF of up to 29%, presumably due to the decreasing number of available amino groups. Successful gelation was proven using the FT-IR spectra of freeze-dried gels (Figure 1a). The stretching peak of the C–O–C at 1100 cm^–1^ and the C=O at 1693 cm^–1^ and the bending vibration of the C–N–C at 1601 cm^–1^ point toward the presence of a cross-linker and PDha-*g*-GTMAC in the hydrogel. Figure 1b exhibits the ^1^H HR-MAS NMR spectrum of the hydrogel highlighting the backbone protons of the cross-linker at 3.68 ppm and the protons on the quaternary ammonium at 3.20 ppm. The latter is in good agreement with ^1^H NMR signals attributed to side-chain quaternary ammonium groups on the spectrum of PEG-*g*-GTMAC, as seen in SI (Figure S2). To quantify the PDha-*g*-GTMAC content in the final hydrogel, we measured the gel content of the hydrogel after gelation, more specifically the weight loss of the hydrogel during dialyses, collected the dialysate, and analyzed it using ^1^H NMR (Figure S5). We noticed around 50% weight loss after the dialyses, which is mainly unreacted cross-linker (PEGDGE). The dialysate contained negligible amounts of PDha-*g*-GTMAC (Figures S6 and S7), and therefore, we determined the PDha-*g*-GTMAC wt % in the hydrogel to about 28 wt %, being in good agreement with the calculation from elemental analysis (32 wt %).

Since the diffusion in gels depends on the microstructure,^51^ the freeze-dried hydrogels were investigated by SEM, and Figure 1c shows an uneven porous internal structure with an average diameter of pores of 10.5 ± 6 μm. The pore size of hydrogels can be regulated by the ionic strength of the aqueous phase, which in turn can be monitored by the degree of swelling.^52^Figure 1d shows that the DS of hydrogels was 47.5 ± 3 in DI water, gradually dropped by salt addition, and reached 11.6 ± 0.7 at 0.5 M aqueous NaCl. This behavior is typical for polyelectrolyte hydrogels due to charge screening by the addition of salt.^53^ Similar effects have been observed in pristine PDha hydrogels despite the polyzwitterionic feature.^41^ In the current work, the net charge of the hydrogel is positive due to the introduced quaternary ammonium side groups, and this contributes to the degree of swelling, also by repulsive Coulombic interactions.^54^ Besides water, the herein described cross-linked PDha-*g*-GTMAC also showed a DS of 30 ± 5 in DMF as a highly polar organic solvent.

To achieve HER, both the photosensitizer and catalyst must meet specific thermodynamic requirements. An organic dye, 1,7,9,10-tetraselenophenoxy PMI, has demonstrated promising PS performance for HER when paired with [Mo_3_S_13_]^2–^, owing to their suitable redox potentials as previously reported.^24^ Moreover, a time-resolved spectroscopic analysis of the molecular components suggests an oxidative quenching mechanism, wherein the triplet excited state of the photosensitizer reduces the catalyst species, facilitating catalytic turnover. However, in addition to thermodynamic considerations, other factors, such as charge separation and surface reactions, can also influence the overall performance. One strategy to address these challenges involves embedding molecular components within a 3D matrix.^55^ Polyampholytic hydrogels, designed here for this purpose, serve as soft matrices and allow for the sequential placement of the components. The dynamic nature of the hydrogel network, characterized by swelling and shrinking, affects the local environment of the catalyst, complicating the characterization of catalytic processes by using advanced techniques such as time-resolved spectroscopy and electrochemical analysis. Consequently, the evaluation of the developed hydrogel primarily focuses on quantifying the evolved amount of H_2_.

Photosensitizer immobilization was realized by immersing the hydrogel in an *N*-hexanoic acid-1,7,9,10-tetraselenophenoxy perylene monoimide (PMI-Se-COOH) solution (0.02 mM) in DMF for 16 h. PMI-Se-COOH was obtained by the modification of 1,7,9,10-tetraselenophenoxy perylene 3,4-monoanhydride (see SI for experimental details) and features a strong absorption peak at 559 nm (Figure S8), which was used for quantification using a calibration curve at different concentrations (Figure S9). To prepare dye solutions, the aqueous solution of PMI-Se-COOH containing stoichiometric amounts of NaOH was first freeze-dried to deprotonate the carboxylic acid on the dye; then, the freeze-dried dye was dissolved in DMF at a given concentration. The use of an excess amount of NaOH was avoided since it led to a unwanted spectral change of PMI-Se-COOH with a new absorption peak at 700 nm and a color change of the solution from purple to green (Figure S10). By immersing in dye solutions at different concentrations such as 0.01, 0.1, and 0.2 mM, light-harvesting soft networks with ca. 0.4, 2.4, and 4.8 wt % of PMI-Se-COOH were obtained. Successful immobilization occurred due to attractive electrostatic interactions between the quaternary ammonium cations and the carboxylate group of the dye. All three gel samples generated in this way were tested as light harvesters in photocatalytic HER reactions.

**Scheme 2:**
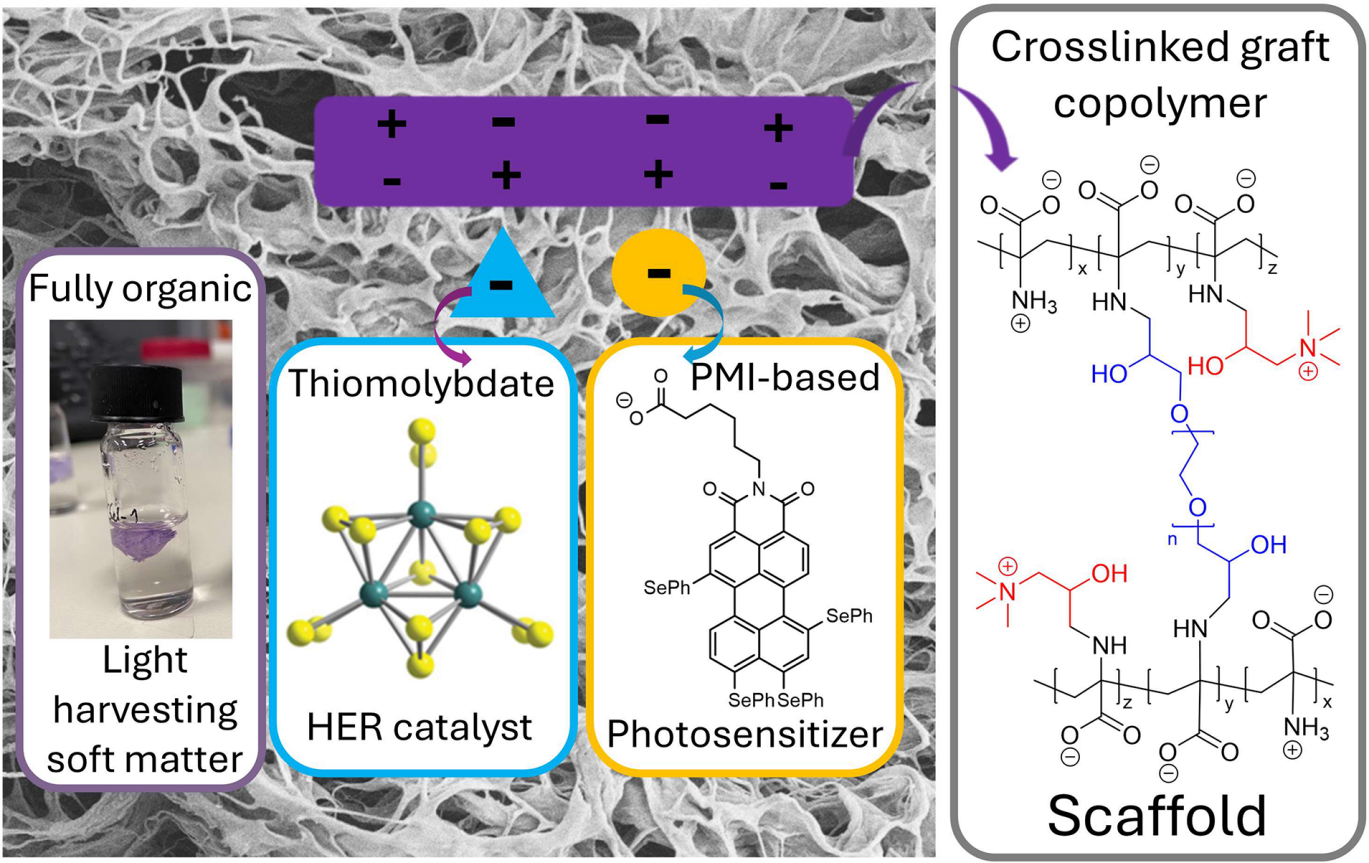
Illustration and Function Principle of the Herein Reported Fully Organic Hybrid Gels for Light-Driven Hydrogen Evolution Reaction (HER)

Next, we combined the PS@PDha-*g*-GTMAC gel with solutions of a thiomolybdate catalyst, [Mo_3_S_13_]^2–^, leading to CAT@PS@PDha-*g*-GTMAC hydrogels due to electrostatic attraction of the catalyst anion by cationic species on the hydrogel, as illustrated in Scheme 2,^27^ and tested the resulting hybrid gels as heterogeneous catalysts for visible light-driven HER in the presence of AA as sacrificial electron donor in water. The gels were placed in 1 M AA solution at pH ≈ 5.5 adjusted by NH_4_OH, and subsequently the thiomolybdate catalyst was added.^56^ After heating for 15 min at 50 °C to promote diffusion of catalyst into the hydrogel, the samples were irradiated with LED light sources in a custom-built photoreactor on a laboratory shaker, and hydrogen production was followed over time using calibrated headspace GC. After 24 h of irradiation, each data point was recorded in triplicate, and the data given are the average amounts of hydrogen detected. During initial tests, we found that hydrogels containing both PS and CAT were able to evolve hydrogen, while gels containing only PS were not active.

**Figure 2:**
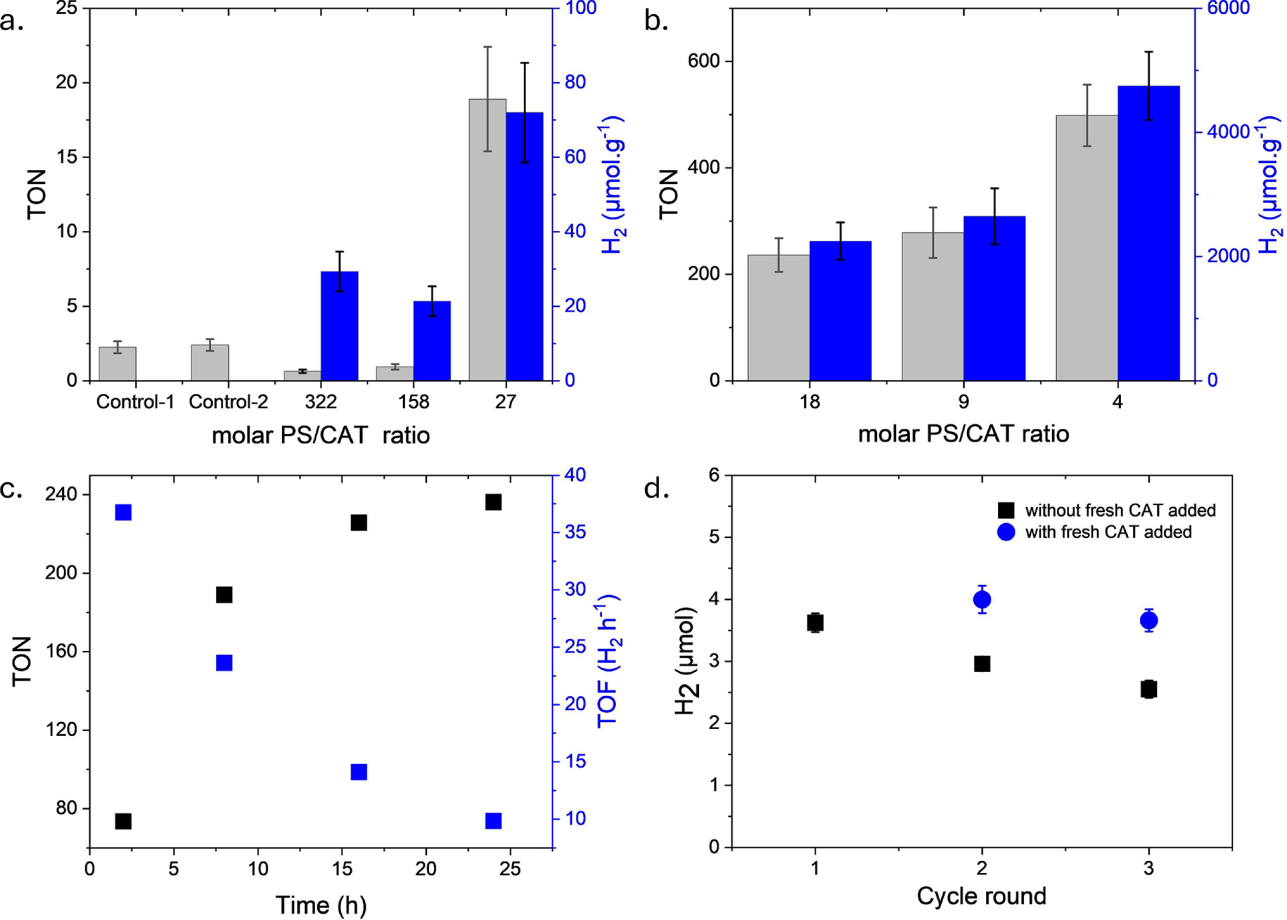
Visible light-driven hydrogen evolution performance in 1 M AA solution for CAT@PS@PDha-*g*-GTMAC hydrogels at varying PS/CAT molar ratios where the turnover number (TON) refers to the moles of hydrogen produced per mole of PS (PMI-Se-COOH), and the turnover frequency (TOF) refers to the TON per hour of irradiation. (A) Effect of PS/CAT molar ratio in the CAT@PS@PDha-*g*-GTMAC hydrogels with 120 ppm catalyst. The control experiments represent the performance in case of no hydrogel; control-1 refers to PS/CAT molar ratio of 187, and control-2 refers to PS/CAT molar ratio of 47. (B) Effect of PS/CAT molar ratio in the CAT@PS@PDha-*g*-GTMAC hydrogels with 1 wt % of PS. (C) Hydrogen production kinetics for CAT@PS@PDha-*g*-GTMAC hydrogels at a PS/CAT ratio of 18. (D) Prospective rounds of hydrogen production using the same hydrogel as used in C; black squares refer to the addition of only fresh AA; blue dots refer to the addition of fresh AA and catalyst before the next round.

We further investigated the effect of various parameters such as the concentrations of PMI-Se-COOH and [Mo_3_S_13_]^2–^ on photocatalytic hydrogen evolution performance (Figure 2). Under optimized PS and CAT concentrations, superior performance was found for the CAT@PS@PDha-*g*-GTMAC gel at a molar PS/CAT ratio of 27 if compared to control experiments using both CAT and PS without polymeric support (Figure 2a). PS@PDha-*g*-GTMAC hydrogels with 0.4, 2.4, and 4.8 wt % of PMI-Se-COOH were combined with [Mo_3_S_13_]^2–^ at a fixed CAT concentration of 120 ppm. Decreasing the PS/CAT ratio from 322 to 158 led to a slight decrease of the turnover number (TON, defined based on moles of PS). On the contrary, a further decrease in the PS/CAT ratio until 27 resulted in a sharp increase of TON, with about 72 ± 13 μmol per gram of PDha-*g*-GTMAC dry gel being produced in the best case (PS/CAT molar ratio of 27) after 24 h of irradiation. One explanation for the decrease at the ratio of PS/CAT of 158 could be an increased influence of back electron transfer from CAT to excited PS molecules, suggesting that different factors may dominate overall hydrogen production under confinement and at different densities of catalytically active building blocks.

As the photocatalytic performance of the herein described system was strongly dependent on the molar PS/CAT ratio, we further varied this by increasing the [Mo_3_S_13_]^2–^ concentration at a constant PS loading of 1 wt % (Figure 2b). This led to an improved H_2_ production performance, with average TONs of 240, 280, and 500 at PS/CAT molar ratios of 18, 9, and 4, respectively, corresponding to 4750 ± 550 μmol/g in H_2_ produced in the latter case. Moreover, the latter case resulted in a PE of 0.03%. The calculation was based on the amount of photons incident to the reaction solution per sample over 24 h under the assumption of full photon absorption. However, for the used hydrogel potential scattering, reflection or transmission of photons was not taken into account. Therefore, the derived PE might underestimate the actual efficiency of the used hydrogel, resulting in potentially even higher photon utilization for the used material.

Noteworthy, the increasing amount of hydrogen produced was directly correlated to the amount of catalyst being present, and therefore, we presume that CAT diffusion is a limiting factor, possibly also because at higher PS loadings hydrophobic interactions between different PS molecules might lead to a lower amount of accessible sites of CAT. With that, the herein described gels with ca. 30% PDha-*g*-GTMAC act as a soft matrix able to immobilize both PS and CAT, leading to a noble metal-free hydrogel device for light-driven HER.

Figure 2c shows the continuous hydrogen production in CAT@PS@PDha-*g*-GTMAC gel at a PS/CAT molar ratio of 18 under visible light irradiation during 24 h. Kinetic studies revealed that the turnover frequency (TOF, defined as TON per hour) gradually decreased with the turnover number reaching toward a plateau. We suppose this decrease was due to the consumption of ascorbic acid because the hydrogel started evolving hydrogen again when a further sacrificial donor was added. Furthermore, we tested the stability of the electrostatic attachment of PMI-Se-COOH toward the PDha-*g*-GTMAC hydrogel under catalytic reaction conditions with the following protocol. The PS@PDha-*g*-GTMAC gel was kept in 1 M AA solution at pH ≈ 5.5 under stirring for 24 h and was then removed, and the catalyst was added into the remaining solution, followed by irradiation for 24 h. We observed no hydrogen production, indicating no significant leaching of PMI-Se-COOH, which was also verified by UV–vis spectroscopy.

Since PS@PDha-*g*-GTMAC hydrogels showed no significant leaching under these conditions, the irradiated hydrogels can be washed with deionized water to remove byproducts, and if necessary, both the catalyst and sacrificial electron donor can be replenished. In this way, we investigated the reusability of such materials in multiple cycles of photocatalytic hydrogen evolution (Figure 2d). To this end, a CAT@PS@PDha-*g*-GTMAC hydrogel with 1 wt % of PMI-Se-COOH and 450 ppm of [Mo_3_S_13_]^2–^ was exposed to additional irradiation for 8 h after this treatment. Without fresh Na_2_[Mo_3_S_13_], we observed a decrease in efficiency to about ∼82% mol H_2_ and around 71% during the next (third) round. We explain this decrease in efficiency by potential leaching of the thiomolybdate catalyst and some catalyst degradation, probably arising from ligand exchange between their terminal disulfides and water.^11^ If a fresh catalyst solution (the same amount as in the hydrogel) is added to the hydrogel prior to the second round of light-driven catalysis, the amount of H_2_ produced increased by a factor of about 1.1. Furthermore, for the third cycle of catalyst addition, we noted that the photocatalytic HER performance was comparable to that of the first round. These results support our hypothesis for reusability of PS@PDha-*g*-GTMAC hydrogels in photocatalytic HER, providing a platform for efficient immobilization of PS/CAT combinations by electrostatic interactions. In our opinion, the straightforward modification and cross-linking of PDha create an attractive platform for light-driven catalysis within hybrid soft matter materials.

## Conclusion

We demonstrate a polyampholyte-based fully organic light-harvesting hydrogel that produces hydrogen under visible light illumination when combined with [Mo_3_S_13_]^2–^ as the HER catalyst and ascorbic acid as the sacrificial electron donor. The chemical cross-linking of modified polydehydroalanine using PEGDGE in combination with an additional modification rendered polyampholytic hydrogels with an excess positive charge, capable of electrostatic immobilization of an organic PS (PMI-Se-COOH) as well as the negatively charged [Mo_3_S_13_]^2–^ catalyst afterward. The resulting hybrid hydrogels exhibited superior catalytic performance compared with their molecularly dissolved counterparts, achieving a hydrogen evolution rate of 198 μmol·g^–1^·h^–1^ with a PS/CAT molar ratio of 4. Notably, this rate was attained at a rather low loading of 0.4% PS and 120 ppm of CAT, further highlighting the potential for sustainable and efficient hydrogen evolution in an aqueous environment. Within 24 h, this leads to an overall 4750 μmol·g^–1^ of H_2_, even outperforming earlier studies comprising [Ru(bpy)_3_]^2+^/PtNP and [Ru(bpy)_3_]^2+^/[Mo_3_S_13_]^2–^, which displayed H_2_ evolution rates of 520 and 3590 μmol·g^–1^, respectively. The hybrid hydrogel presented herein demonstrates a significant enhancement in H_2_ production, using an established reactor setup for quantification and illumination, ensuring a robust evaluation of performance across different systems. We show that the designed hydrogels were reusable multiple times by sequential addition of the sacrificial agent and eventually also adding fresh CAT, allowing to exchange degraded catalytic sites within the soft matter matrix. In our opinion, this can be a starting point for future noble metal-free systems for artificial photosynthesis.

## Supporting Information Available

Experimental details for the synthesis of PMI-Se-COOH, additional discussion for the characterization of PDha-*g*-GTMAC, ^1^H NMR spectra of P*t*BAMA, P(Dha-*co*-AMA), and PDha-*g*-GTMAC, ^13^C NMR spectra of PDha-*g*-GTMAC and GTMAC, list of DoF values for PDha-*g*-GTMAC, FT-IR spectra of PDha-*g*-GTMAC, ^1^H NMR spectrum of dialysate sample taken during dialysis after cross-linking, calibration curve showing PDha-*g*-GTMAC content of different integration ratios for the standard mixture of PEGDGE and PDha-*g*-GTMAC, quantitative ^1^H NMR spectrum of the mixture of PEGDGE and PDha-*g*-GTMAC, absorption spectrum of PMI-Se-COOH, calibration curve showing optical density at 559 nm of different concentrations of PMI-Se-COOH, ^1^H, ^13^C, and ^77^Se NMR spectra of PMI-Se-COOH (PDF)
