# Supplementary material for: Noble Metal-Free Light-Driven Hydrogen Evolution Catalysis in Polyampholytic Hydrogel Networks
Source: ACS Appl Mater Interfaces. 2024 May 3;16(19):24796–805. doi: 10.1021/acsami.4c04045 (PMC11103662; doi:10.1021/acsami.4c04045)
Supplement: Supplementary file 1 — am4c04045_si_001.pdf [file am4c04045_si_001.pdf]

Supporting Information

Noble metal-free light-driven hydrogen evolution catalysis in polyampholytic hydrogel networks

Tolga Ceper[a,b,c], *Daniel Costabel*[a], *Daniel Kowalczyk*[d], *Kalina Peneva*[a,b,c], *Felix H. Schacher**[a,b,c]

[a] Institute of Organic Chemistry and Macromolecular Chemistry, Friedrich Schiller University Jena, Humboldtstraße 10, D-07743 Jena, Germany. E-mail: felix.schacher@uni-jena.de

[b] Jena Center for Soft Matter (JCSM), Friedrich Schiller University Jena, Philosophenweg 7, D-07743 Jena, Germany

[c] Center for Energy and Environmental Chemistry Jena (CEEC), Friedrich Schiller University Jena, Philosophenweg 7a, 07743 Jena, Germany

[d] Institute of Chemical Engineering, Ulm University, Albert-Einstein-Allee 11, 89081, Ulm, Germany

Synthesis of *N*-hexanoic acid-1,7,9,10-tetraselenophenoxy perylene monoimide (PMI-Se-COOH)

A mixture of 1,7,9,10-tetraselenophenoxy perylene 3,4-monoanhydride (100 mg, 0.11 mmol), zinc acetate dihydrate (23 mg, 0.11 mmol), 6-aminohexanoic acid (70 mg, 0.53 mmol) and immidazole (3 g, 44 mmol) was melted and stirred at 120°C and the reaction mixture was monitored by TLC. After 10 minutes, the starting material was consumed and the reaction mixture was allowed to cool to room temperature. The solid residue was dissolved in DCM and 1 M aqueous HCl. The organic layer was separated, dried over Na_2SO_4 and the solvent was evaporated under reduced pressure. The crude product was purified by column chromatography (DCM: MeOH 95:5).

Yield: 89 mg, 79%

^{1}H-NMR ($CDCl_3$, 300 MHz): 8.61 (d, J = 7.9 Hz, 1H), 8.39 (s, 1H), 7.93 (d, J = 7.9 Hz, 1H), 7.74 (d, J = 8.1 Hz, 1H), 7.67 (d, J = 8.1 Hz, 1H), 7.60 (s, 1H), 7.59-7.51 (m, 4H), 7.41-7.27 (m, 12H), 7.23-7.14 (m, 2H), 4.12 (t, J = 7.5 Hz, 1H), 2.35 (t, J = 7.5 Hz, 2H), 1.82-1.60 (m, 4H), 1.51-1.39 (m, 2 H) ppm.

$^{13}C^{1}$-NMR ($CDCl_3$, 100 MHz): 177.9, 164.0, 163.6, 138.7, 137.0, 136.9, 135.9, 135.8, 135.8, 135.7, 135.5, 135.2, 134.4, 134.4, 134.1, 133.1, 133.0, 132.5, 131.8, 131.3, 130.9, 130.1, 130.0, 129.9, 129.9, 129.5, 129.2, 128.7, 128.5, 128.3, 127.8, 126.2, 125.9, 40.3, 33.7, 29.8, 26.7, 24.5 ppm.

^{77}Se-NMR ($CDCl_3$, 76 MHz): 449.6, 447.8, 424.1, 421.7 ppm.

APCI-MS pos.: 1057.952 ($M+H^+$), 1057.946 (calc.).

Additional discussion

Characterization of PDha-*g*-GTMAC

Polymer backbone and pendant groups were both assigned in ^{1}H NMR spectra of P(Dha-*g*-GTMAC). (Fig.S2) Backbone protons present between 2.80 and 1.90 ppm in accordance with the spectrum of non-functionalized PDha.[2] The signals of methyl protons $–N(CH_3)_3$ are visible between 3.19 and 2.96 ppm, giving a peak a 3.11 ppm, which is overlaid by $–N–CH_2–$ protons of the side-chain.

In the ^{13}C-NMR spectrum of P(Dha-*g*-GTMAC), carbonyl carbon C=O was clearly visible at 180 ppm, which is in accordance with non-functionalized PDha, while backbone carbons were overlaid with side-chain carbons (Fig. S3). Backbone carbons of non-functionalized PDha were present at 47 ppm for non-substituted and at 65 ppm for double-substituted carbon.[2] P(Dha-*g*-GTMAC) shows a strong peak at 54 ppm for three methyl carbons $–N(CH_3)_3$ and a broad peak at 70 ppm for –C–OH which originate from the side-chains in agreement with the NMR spectrum of the corresponding epoxide. The epoxide signals at 46 and 45 ppm disappear after modification.

The FT-IR spectrum to verify the P(Dha-*g*-GTMAC) synthesis is illustrated in Figure S4. The spectrum included adsorption increase at 1601 cm^{-1}, corresponding to the C-N-C bending vibration of the GTMAC branch. The C=O stretching vibration of the carbonyl group from the PDha, occurring at 1693 cm^{-1}, is an invariant peak which should be the same for both PDha and P(Dha-*g*-GTMAC) polymers. The adsorption between 3300 and 2800 cm^{-1} was attributed to the C-H stretching vibration of the CH_2 or CH_3 groups. Absorption due to C-N vibrations occurs between 1220 and 1020 cm^{-1}, where an increase at 1099 cm^{-1} appeared arising from tertiary amine testifying attachment of GTMAC. Finally, a broad peak between 3685 and 3014 cm^{-1} represented the combined O-H stretching vibration and N-H stretching vibration, which

was increased after GTMAC attachment due to the formation of new –OH bond resulting from epoxide ring-opening.

Figures and tables

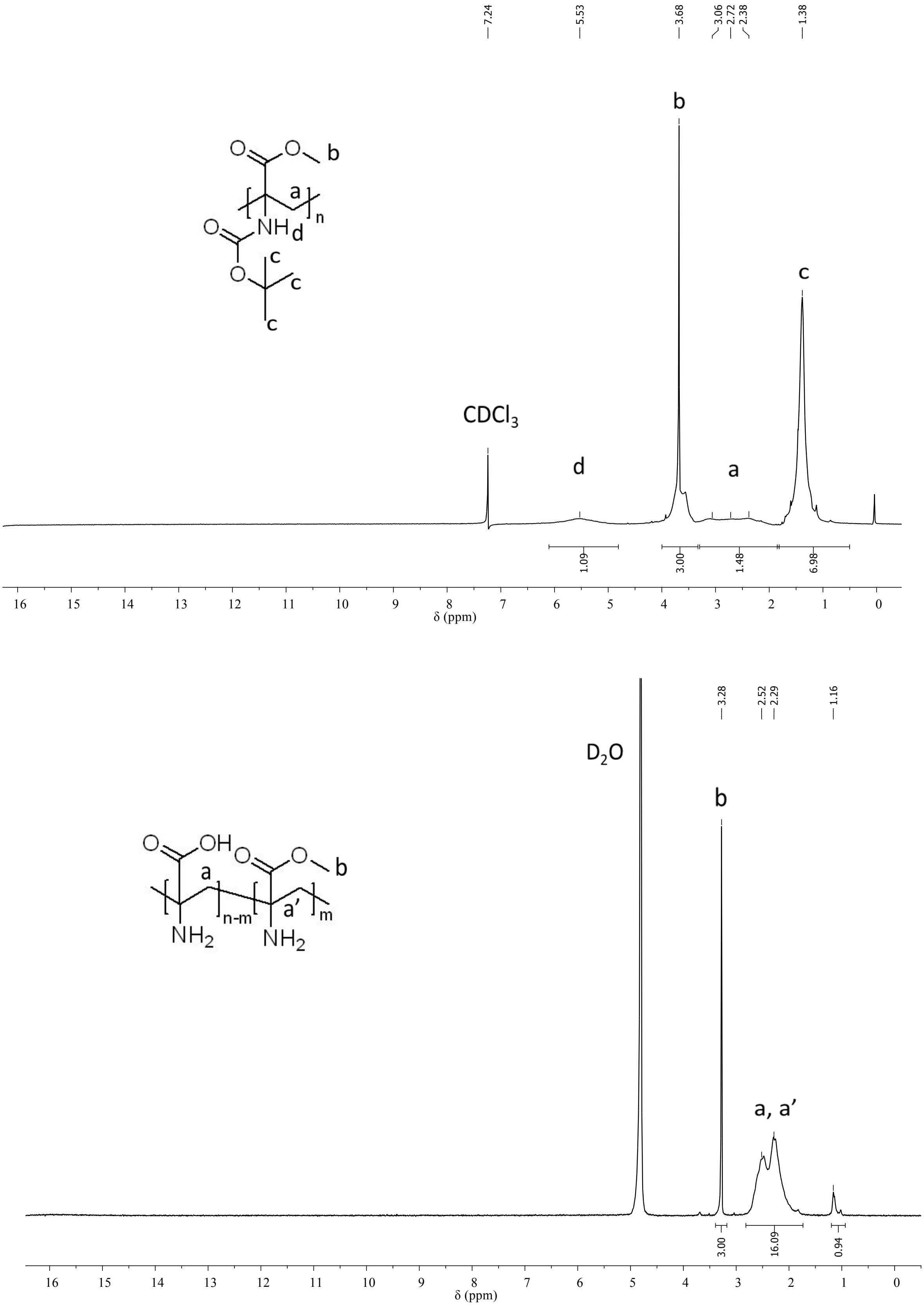

Figure S1. ^{1}H NMR of P*t*BAMA in $CDCl_3$ (top) and corresponding P(Dha-*co*-AMA) in D_2O + NaOD (bottom, n/m is 9, the appearance of H signal of COOH can not be observed due to the presence of D_2O) after deprotection reaction.

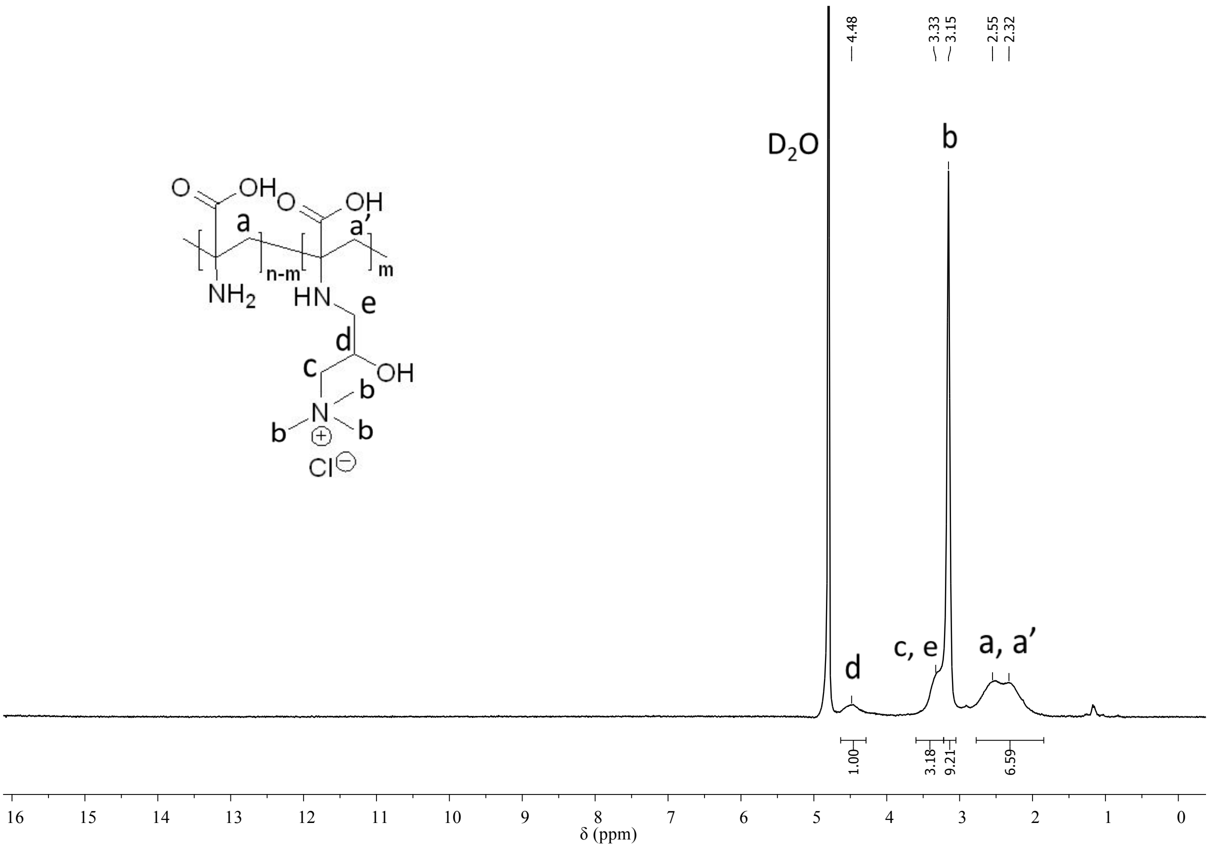

Figure S2. ^{1}H NMR of PDha-*g*-GTMAC in D_2O + NaOD.

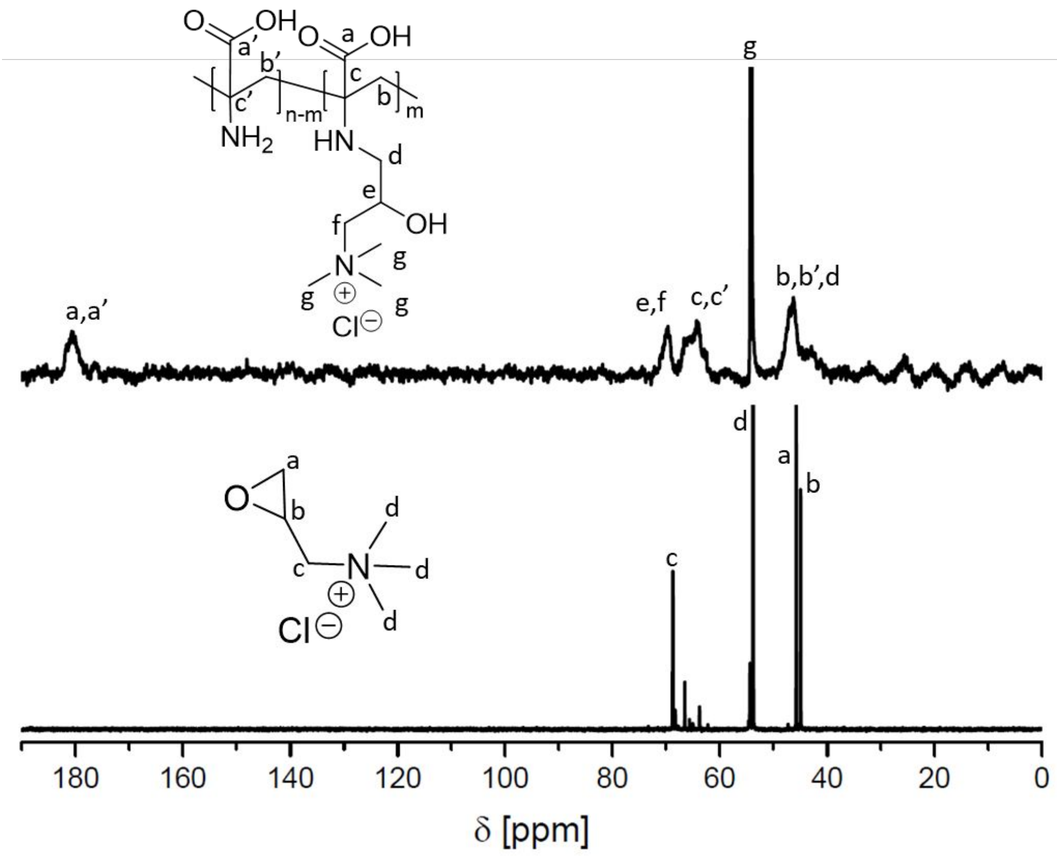

Figure S3. ^{13}C NMR before and after modification of PDha with GTMAC in D_2O + NaOD.

Table S1. Reaction parameters for the modification of PDha-*co*-AMA with GTMAC and the corresponding degrees of functionalization.

P(Dha-*co*-AMA)-to-GTMAC	Duration	DoF
1:10	7 h	99%
1:1.5	4 days	48%
1:1	5 days	29%
1:1	4 days	23%

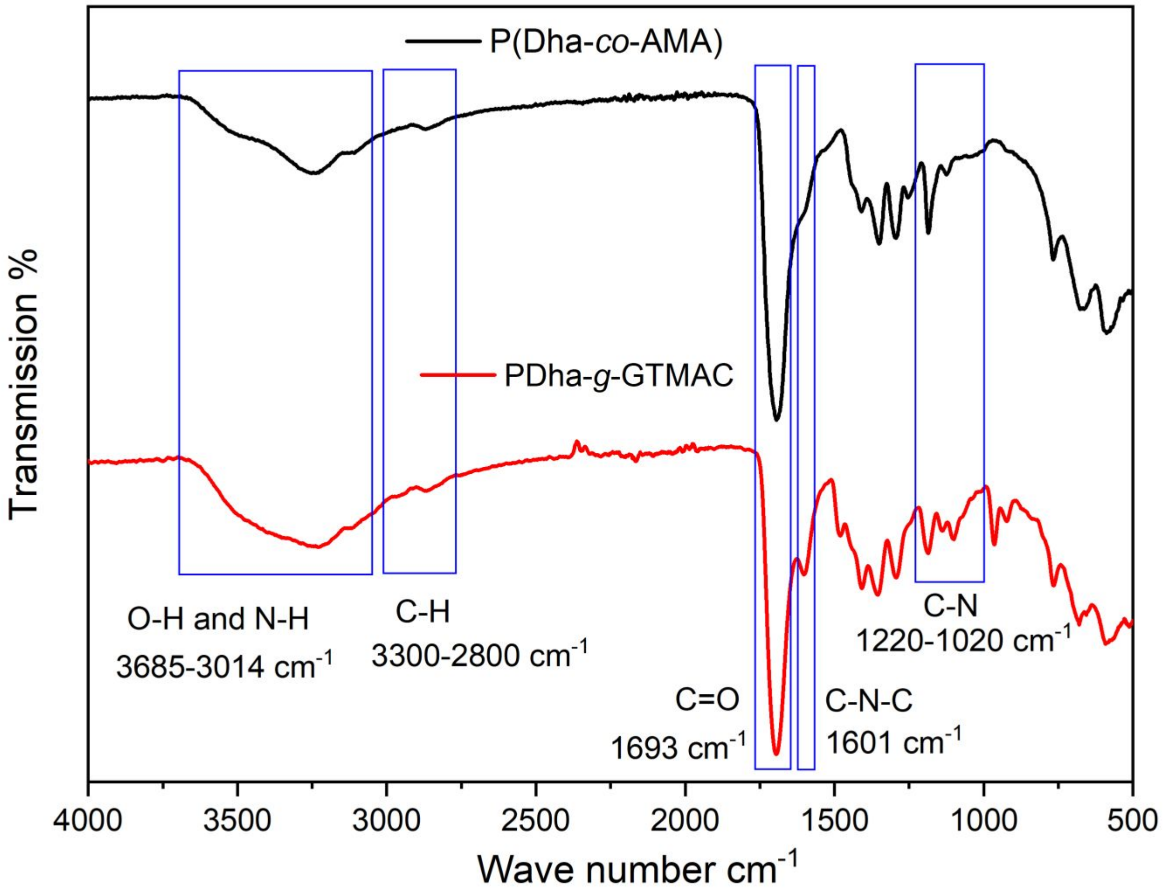

Figure S4. Comparison of the FTIR spectra before and after modification of P(Dha-*co*-AMA) with GTMAC.

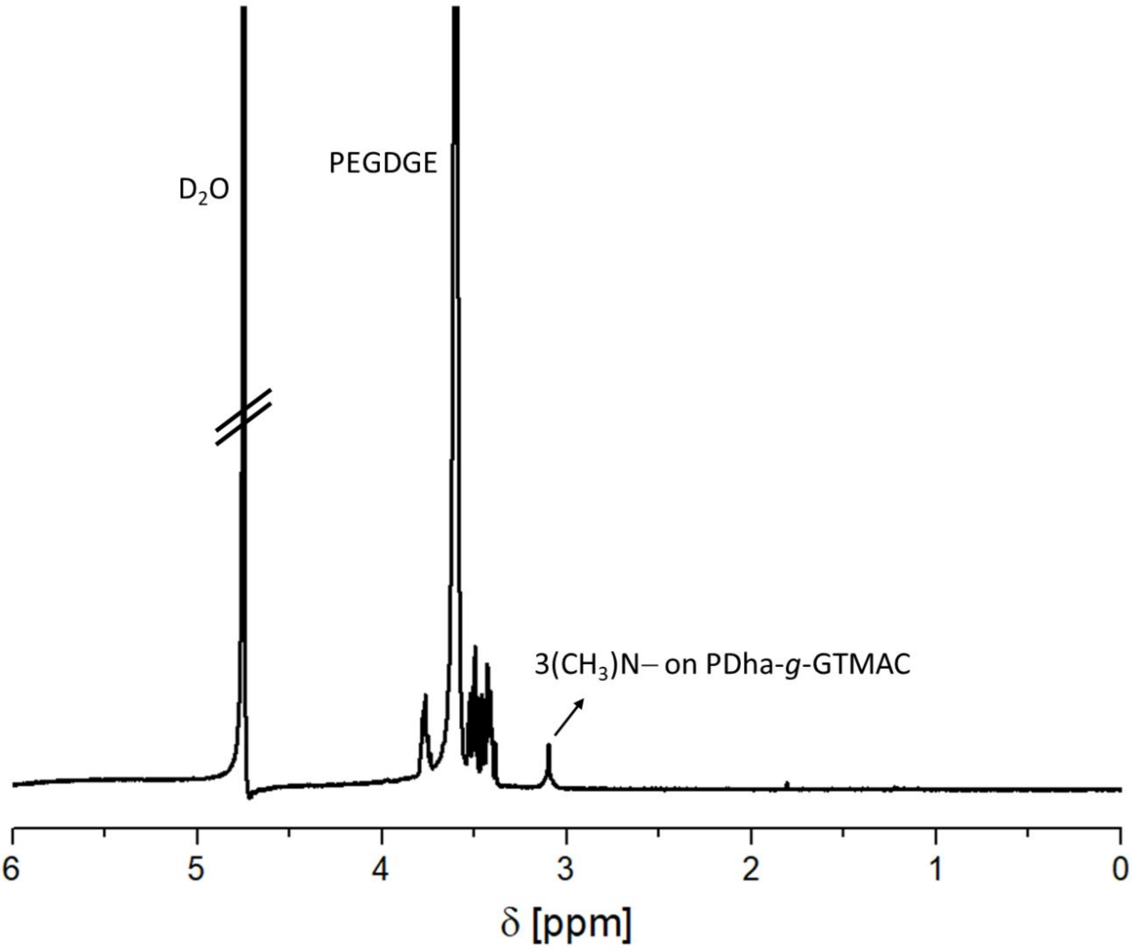

Figure S5. ^1H NMR spectrum of dialysate collected from PDha-*g*-GTMAC and PEGDGE crosslinking.

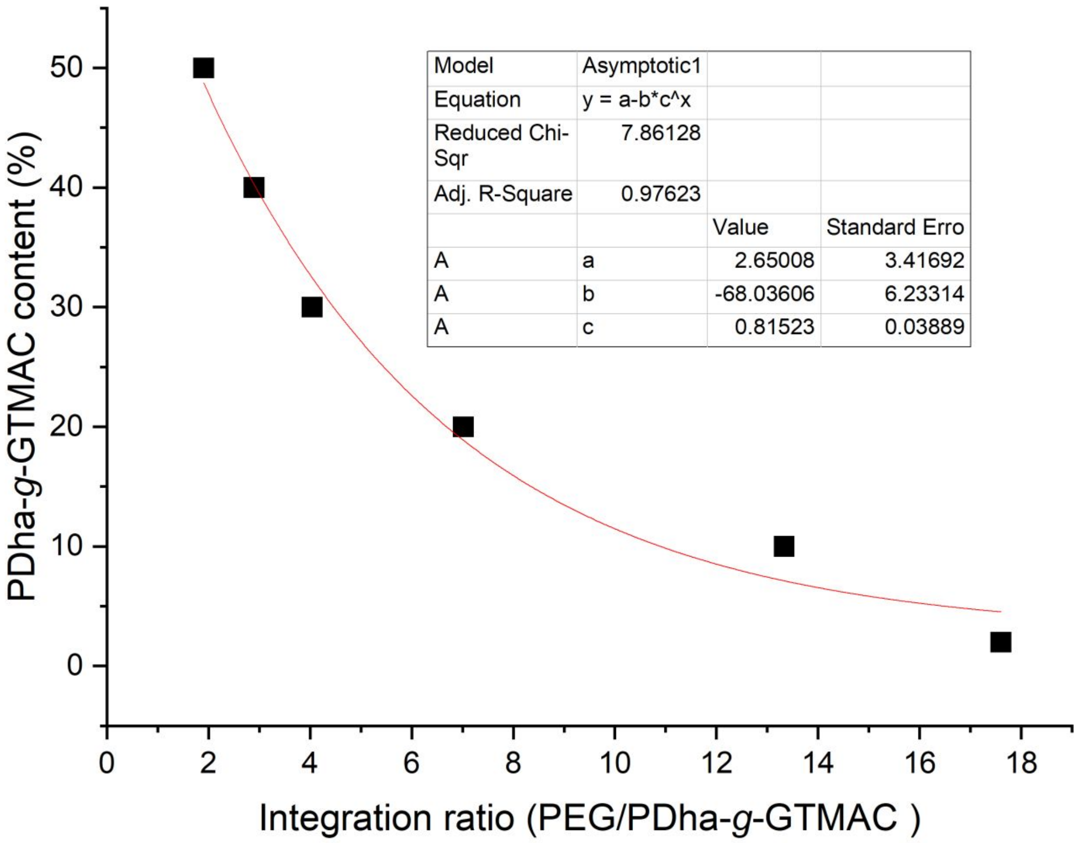

Figure S6. Calibration curve showing PDha-*g*-GTMAC content of different integration ratios for the standard mixture of PEGDGE and PDha-*g*-GTMAC (3.73-3.54 ppm was integrated for PEGDGE, 3.3-1.5 ppm was for PDha-*g*-GTMAC on the quantitative ^{1}H NMR spectrum in Figure S7).

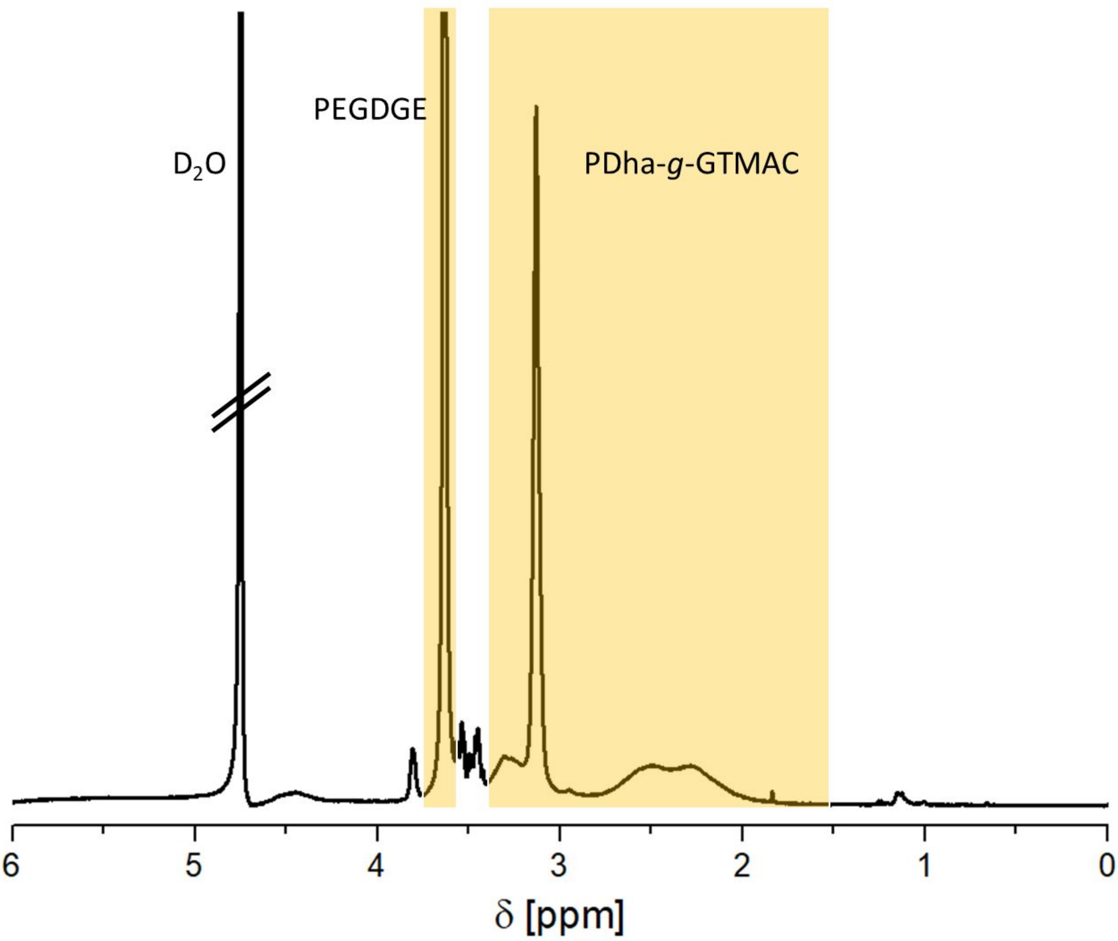

Figure S7. ^{1}H NMR spectrum of 50:50 PEGDGE and PDha-*g*-GTMAC mixture and corresponding peak area used for the calibration curve.

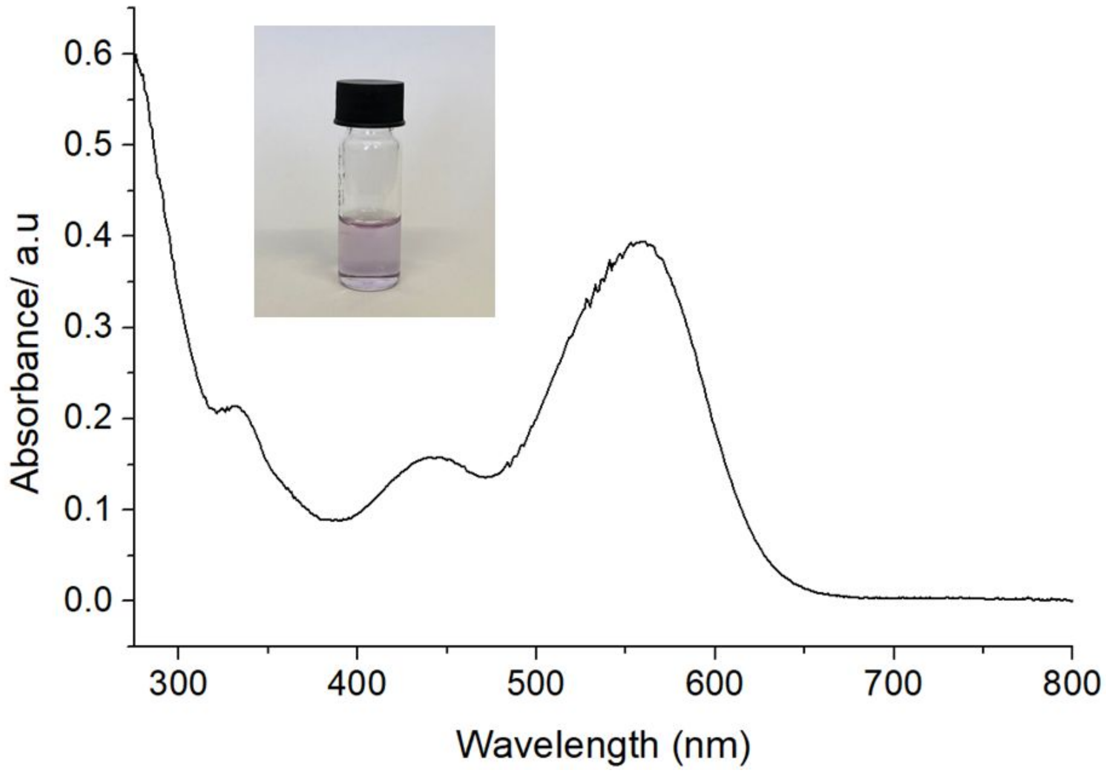

Figure S8. Absorption spectrum of PMI-Se-COOH in DMF (20 μM).

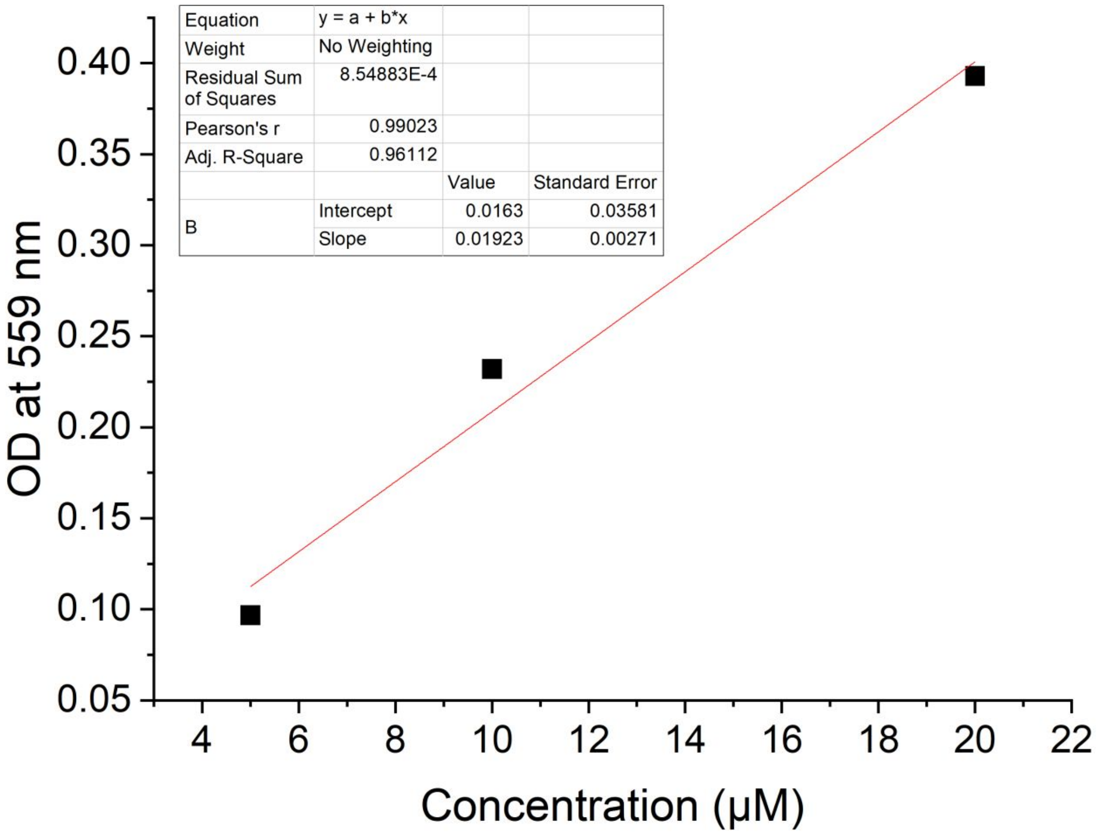

Figure S9. Calibration curve showing optical density at 559 nm of different concentration of PMI-Se-COOH in DMF.

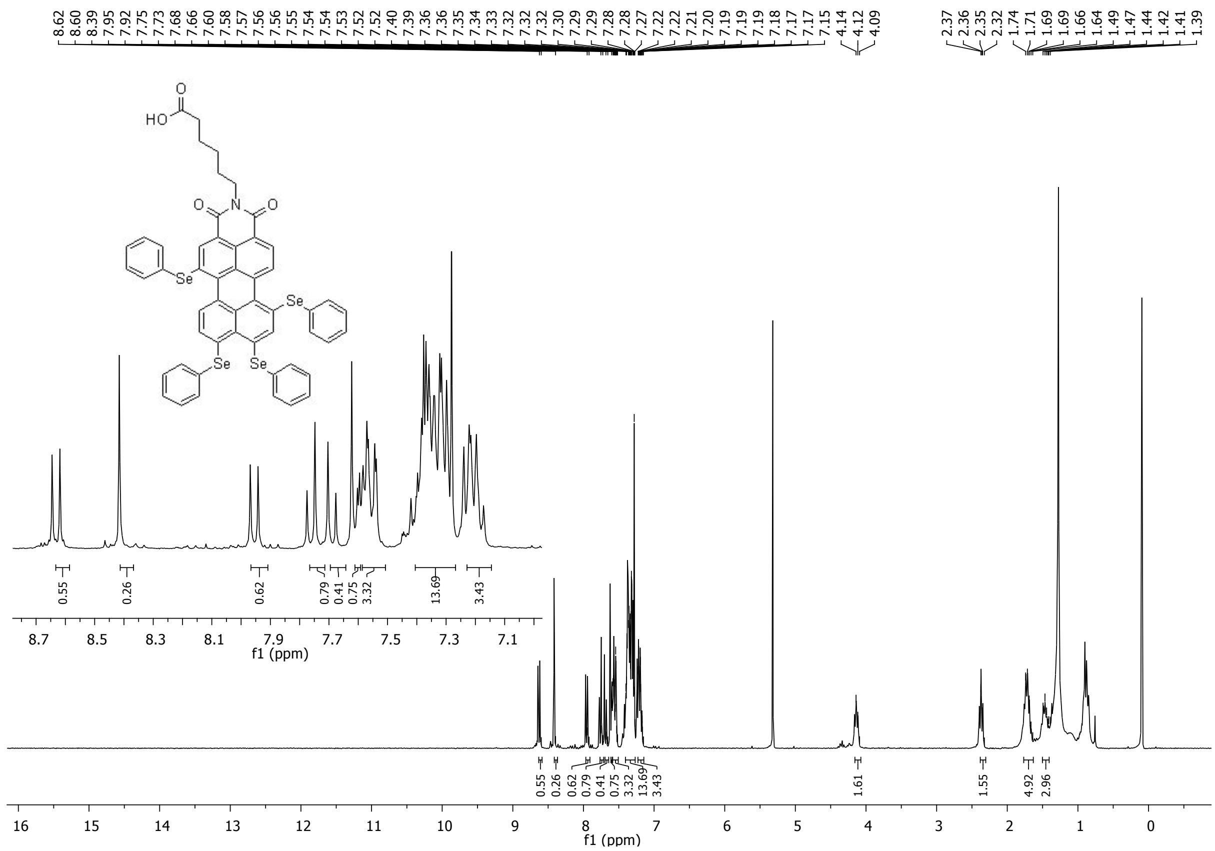

Figure S10. ^1H NMR of PMI-Se-COOH ($CDCl_3$, 300 Mhz).

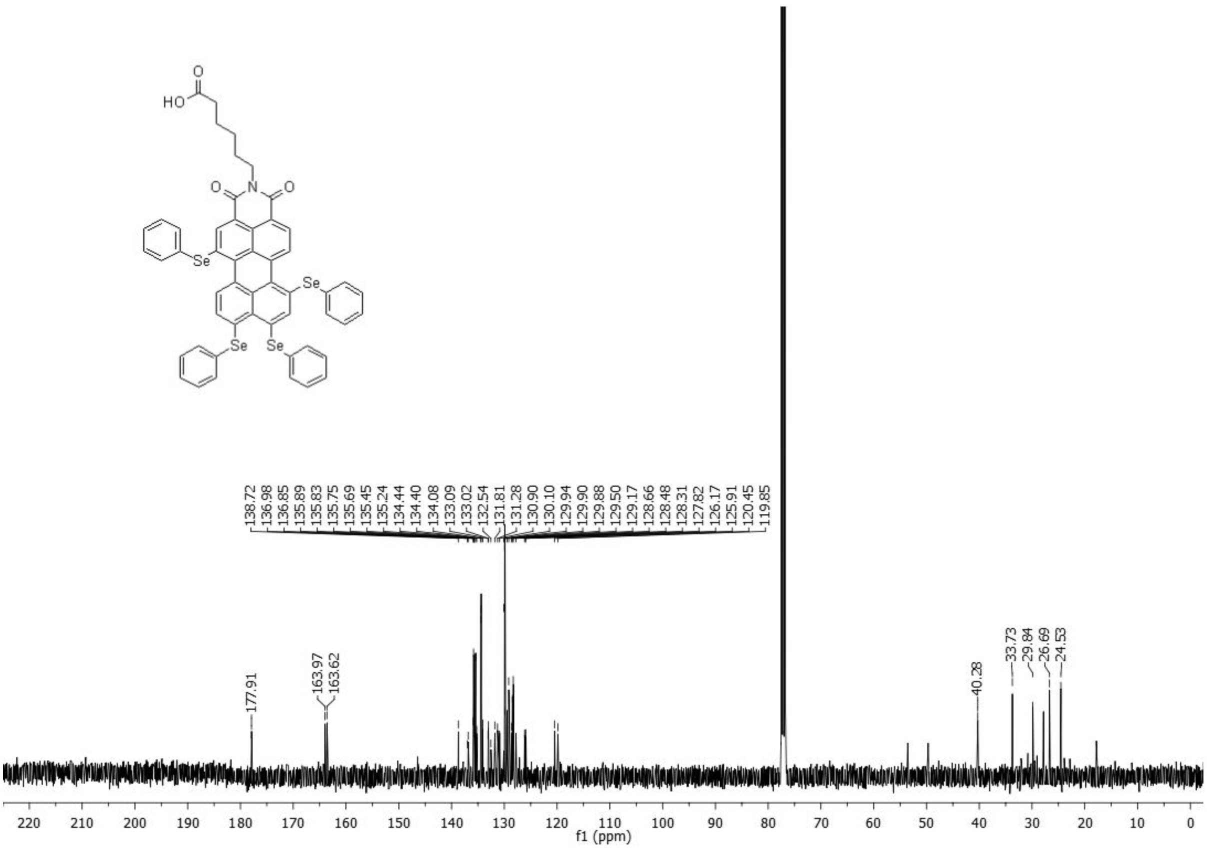

Figure S11. ^{13}C NMR[1] of PMI-Se-COOH ($CDCl_3$, 100 Mhz).

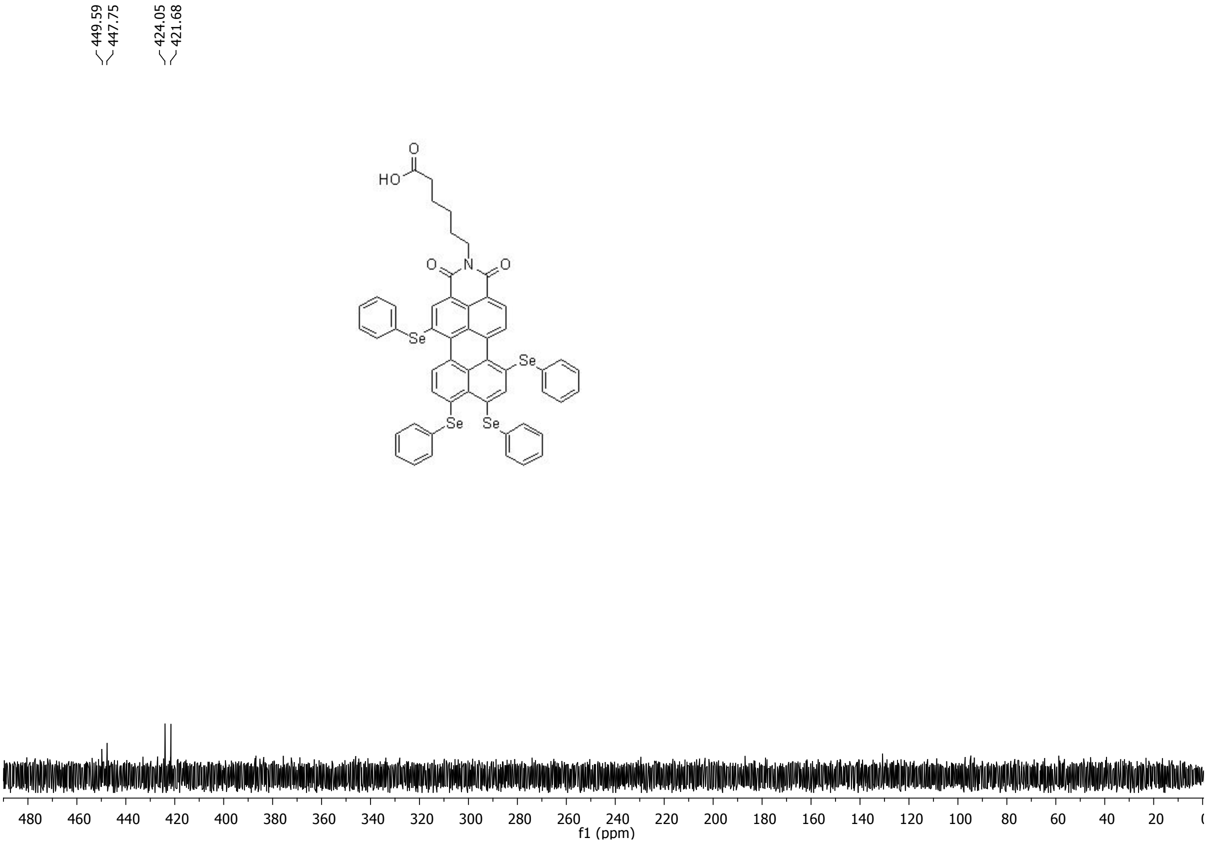

Figure S12. ^{77}Se NMR of PMI-Se-COOH ($CDCl_3$, 76 Mhz).

References

1. Ali, Q.; Ahmad, N.; Ashraf, M.; Rashid, M.; Schacher, T., Shake Table Tests on Single-Story Dhajji Dewari Traditional Buildings. *Int J Archit Herit* **2017,** *11* (7), 1046-1059.

2. Max, J. B.; Pergushov, D. V.; Sigolaeva, L. V.; Schacher, F. H., Polyampholytic graft copolymers based on polydehydroalanine (PDha) - synthesis, solution behavior and application as dispersants for carbon nanotubes. *Polym Chem-Uk* **2019,** *10* (23), 3006-3019.
